# Natural and Synthetic Xanthone Derivatives Counteract Oxidative Stress via Nrf2 Modulation in Inflamed Human Macrophages

**DOI:** 10.3390/ijms232113319

**Published:** 2022-11-01

**Authors:** Marialucia Gallorini, Simone Carradori, Diana I. S. P. Resende, Luciano Saso, Alessia Ricci, Andreia Palmeira, Amelia Cataldi, Madalena Pinto, Emília Sousa

**Affiliations:** 1 Department of Pharmacy, University “G. d’Annunzio” Chieti-Pescara, Via dei Vestini 31, 66100 Chieti, Italy; 2 Laboratory of Organic and Pharmaceutical Chemistry, Department of Chemical Sciences, Faculty of Pharmacy, University of Porto, 4050-313 Porto, Portugal; 3 CIIMAR–Interdisciplinary Centre of Marine and Environmental Research, Avenida General Norton de Matos, S/N, 4450-208 Matosinhos, Portugal; 4 Department of Physiology and Pharmacology “Vittorio Erspamer”, Sapienza University of Rome, P. le Aldo Moro 5, 00185 Rome, Italy

**Keywords:** Nrf2, inflammation, oxidative stress, xanthones, macrophages, mangostins

## Abstract

Natural products have attracted attention due to their safety and potential effectiveness as anti-inflammatory drugs. Particularly, xanthones owning a unique 9*H*-xanthen-9-one scaffold, are endowed with a large diversity of medical applications, including antioxidant and anti-inflammatory activities, because their core accommodates a vast variety of substituents at different positions. Among others, α- and γ-mangostin are the major known xanthones purified from *Garcinia mangostana* with demonstrated anti-inflammatory and antioxidant effects by in vitro and in vivo modulation of the Nrf2 (nuclear factor erythroid-derived 2-like 2) pathway. However, the main mechanism of action of xanthones and their derivatives is still only partially disclosed, and further investigations are needed to improve their potential clinical outcomes. In this light, a library of xanthone derivatives was synthesized and biologically evaluated in vitro on human macrophages under pro-inflammatory conditions. Furthermore, structure–activity relationship (SAR) studies were performed by means of matched molecular pairs (MMPs). The data obtained revealed that the most promising compounds in terms of biocompatibility and counteraction of cytotoxicity are the ones that enhance the Nrf2 translocation, confirming a tight relationship between the xanthone scaffold and the Nrf2 activation as a sign of intracellular cell response towards oxidative stress and inflammation.

## 1. Introduction

Over the last decades, natural compounds have attracted strong interest, not only for their wide range of pharmacological applications but also for the possibility of chemical modification in order to improve their pharmacodynamics or pharmacokinetics. Among these molecules, the heat-stable xanthone scaffold provides advantageous therapeutic effects in different pathological conditions leading researchers to better investigate their potential [1,2]. The core nucleus is represented by the xanthene-9-one, which is characterized by different substitution patterns to elicit a wide range of biological activities, including antioxidant, anti-inflammatory, antimicrobial, and anti-cytotoxic responses. The antioxidant and anti-inflammatory effects have recently emerged as the most relevant, mainly for the treatment of skin inflammatory diseases [3]. However, the main mechanism of action of xanthones and their derivatives is still only partially disclosed. It has been reported that the biological effects of the xanthone core nucleus might be related to the modulation of various pro-inflammatory and anti-inflammatory cytokines as a sign of the recruitment of immune cells. In parallel, nuclear factor kappa-light-chain-enhancer of activated B cells (NF-κB), mitogen-activated protein kinase (MAPK), inducible cyclooxygenase (COX-2), and vascular endothelial growth factor (VEGF)-related signaling pathways are also involved [4].

The most famous natural xanthones are mangostins isolated as secondary metabolites from the mangosteen fruit (*Garcinia mangostana* L.), namely α- and γ-mangostin [5,6]. α-Mangostin is a prenylated xanthonoid endowed with wide-ranging biological effects from anticancer to anti-inflammatory properties. Its key structural features and safety have been investigated [7], although its low oral bioavailability and poor brain penetration have made finding a proper therapeutic application challenging. However, these limitations could be partially overcome by using specific carrier systems [8]. Conversely, γ-mangostin, isolated from the fruit hull, has been only marginally investigated despite it only differing slightly from the previous one (7-OH instead of 7-OCH_3_) [9].

The nuclear factor erythroid 2-related factor 2 (Nrf2) is essential for protection against oxidative/xenobiotic stresses and has been widely known to be activated in immune cells to attenuate inflammation [10]. Previous studies show that Nrf2 exerts its anti-inflammatory effects in bacterial lipopolysaccharide (LPS)-treated macrophages interfering with the Toll-like receptor 4 (TLR4)-induced NF-kB signaling pathway [11]. The relationship between α-mangostin and Nrf2 activation has been demonstrated both in vitro and in vivo in different disease models. In a model of hydrogen peroxide (H_2_O_2_)-stressed RPE cells (age-related macular degeneration model), reactive oxygen species (ROS) generation and malondialdehyde (MDA) production were suppressed, whereas Nrf2-related antioxidant enzymes such as superoxide dismutase (SOD) and glutathione peroxidase (GPX), as well as the reduced glutathione (GSH) content, were increased. These effects have been related to the mangostin-induced Nrf2 nuclear translocation leading to the up-regulation of heme oxygenase-1 (HO-1), a potent antioxidant enzyme [12]. In other work, it was demonstrated that the administration of α-mangostin attenuated lipopolysaccharide/D-galactosamine (LPS/D-GalN) induced acute liver pathological injury in mice by activating Nrf2 and HO-1 and thus enhancing the antioxidant cell defense towards inflammation generated through the TLR4-related signaling. The authors also observed a decrease in specific pro-inflammatory biomarkers such as cytokines, including tumor necrosis factor (TNF-α), interleukin-1β and 6 (IL-1β, IL-6), and the recovery of hepatic GSH, SOD, and catalase activities [13].

The present work aims at evaluating newly synthesized xanthone derivatives in terms of biological activity and Nrf2 activation under pro-inflammatory conditions. With these aims, a model of LPS-stimulated macrophages was established, and xanthone derivatives were administered to measure cell metabolic activity, cytotoxicity, and Nrf2 nuclear translocation. Then, the biological results were analyzed to build a QSAR (Quantitative Structure–Activity Relationship) model to identify structural features influencing activity/potency within this large library of synthetic and natural xanthones.

## 2. Results and Discussion

**Scheme 1:**
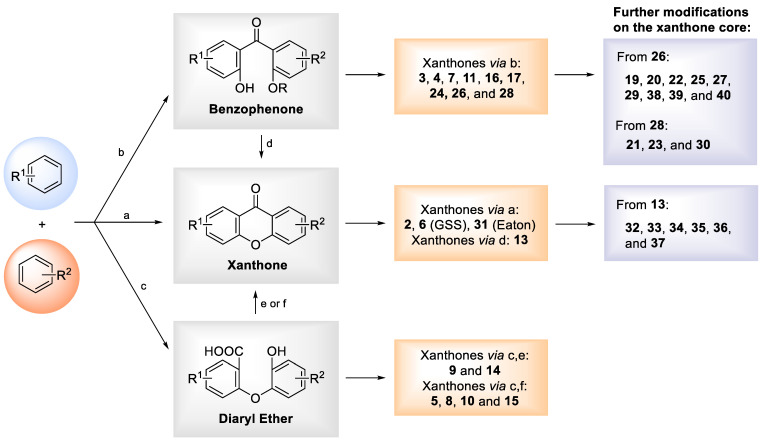
Methodologies used for the synthesis of xanthones **2**–**11**, **13**–**37**, **39**, and **40**. Adapted from [15].

Xanthones **2**–**11**, **13**–**37**, **39**, and **40** (Table 1) were previously synthesized in our laboratory in the scope of other projects via three traditional methods (Scheme 1) [14,15,16,17,18,19,20,21,22,23,24,25,26,27,28,29,30]: condensation of salicylic acid or salicylic ester with a phenol derivative (a), via benzophenone (b), or via diphenyl ether intermediates (c).

Compounds **2**, **6** (Grover, Shah, and Shah), and **31** (Eaton’s reagent) were synthesized via (a), while compounds **3**, **4**, **7**, **11**, **16**, **17**, **24**, **26**, and **28** were obtained via (b). The compound **13** was obtained through a dehydrative process (d) from commercial 2,2′,4,4′-tetrahydroxybenzophenone. The diaryl process led to the synthesis of compounds **9** and **14** via (e) and the compounds **5**, **8**, **10**, and **15** via (f), respectively. Further structural modifications were performed on compounds **13**, **26**, and **28** in order to extend the structural diversity of this library of compounds as previously reported by other groups [23,28,31]. 1,7-Dihydroxyxanthone (**12**) was previously isolated from *Cratoxylum maingayi* (Guttiferae) [15].

**Scheme 2:**
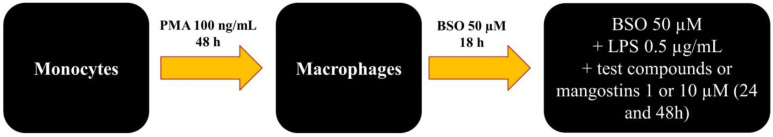
In vitro experimental model used for the screening of xanthone derivatives **1**–**40** and controls (mangostins). Undifferentiated human monocytes (CRL-9855™) were stimulated with 100 ng/mL of PMA (phorbol-12-myristate-13-acetate) for 48 h to obtain differentiated macrophage-like cells. Next, macrophages were pre-incubated with 50 µg/mL of BSO (L-buthionine sulfoximine) for 18 h. After that, cells were stimulated with 0.5 µg/mL of LPS (Lipopolysaccharide) and exposed in parallel to BSO or xanthone derivatives (test compounds) or mangostins for up to 48 h.

To investigate whether the polyphenolic and aminated synthesized xanthone derivatives have biological activity in an inflamed environment, the compounds were tested on LPS-stimulated macrophages. In parallel, cells were pre-incubated and exposed to BSO to trigger oxidative stress and stimulate a sustained Nrf2 activation, as reported in previous studies [32] and shown in Scheme 2. When the LPS and the BSO were added, macrophage metabolic activity dramatically increased (160% of metabolically active cells compared to untreated cells, as a sign of activation. For comparison, macrophages were also exposed to *N*-acetylcysteine (NAC) (5 and 10 mM,), a widely known antioxidant capable of decreasing Nrf2 activation in macrophages under oxidative stress conditions [32] and α- and γ-mangostins. Since mangostins, as natural xanthones, represent the ideal controls for the newly synthesized xanthone derivatives, only data after mangostin exposure are shown.

**Figure 1:**
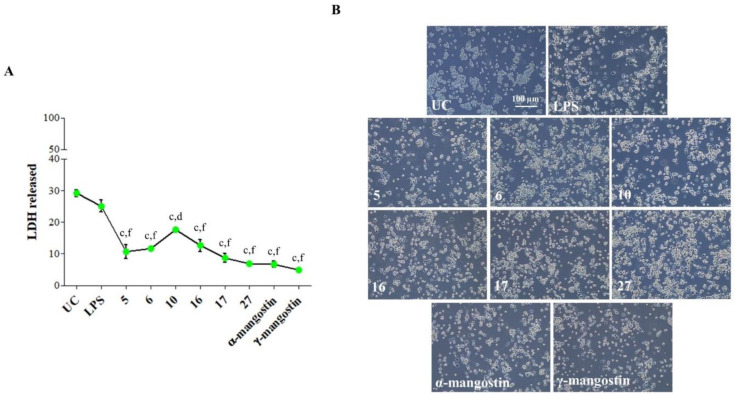
Cytotoxicity occurrence in inflamed macrophages in the presence of compounds **5**, **6**, **10**, **16**, **17**, **27**, and mangostins after 24 h of exposure. (**A**) The trend line graph shows the percentage of LDH released from cells in all the experimental conditions. Values are normalized on 3-(4,5-dimethylthiazol-2-yl)-2,5-diphenyltetrazolium bromide (MTT) data obtained from the same experiment. (**B**) Images from a representative experiment. UC = untreated cells (cells pre-incubated with BSO for 18 h). LPS = cells stimulated with 0.5 µg/mL LPS alone. The pure UC (cells exposed to complete RPMI for the entire experimental procedure) was set as 1 (not shown). c = *p* < 0.0001 between samples and UC; d = *p* < 0.01 and f = *p* < 0.0001 between samples and LPS. Representative images from experiments were obtained by optical microscopy (phase-contrast, magnification 100×).

**Figure 2:**
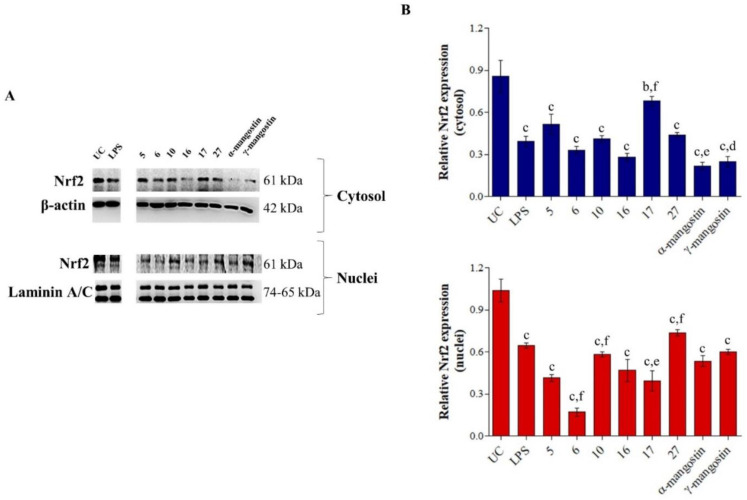
Nrf2 protein expression in cytosols and nuclei of inflamed macrophages in the presence of compounds **5**, **6**, **10**, **16**, **17**, **27**, and mangostins after 24 h of exposure. (**A**) Western blot analysis of Nrf2 in cytosolic and nucleic protein fractions. β-actin and laminin A/C were used as loading controls. (**B**) Bar graphs display densitometric values normalized using the loading controls. UC = untreated cells (cells pre-incubated with BSO for 18 h). LPS = cells stimulated with 0.5 µg/mL LPS alone. b = *p* < 0.001 and c = *p* < 0.0001 between samples and UC; d = *p* < 0.01, e = *p* < 0.001, and f = *p* < 0.0001 between samples and LPS.

When α-mangostin was added at 24 h, the cell metabolic activity significantly increased both at 1 and 10 µM with respect to the sample in the presence of LPS and BSO, which was chosen for 100% of the experimental procedure. This trend maintained after 48 h and was even greater in the presence of γ-mangostin (Table 2 and Table 3). To verify if the increase in metabolic activity and, thus, the macrophage activation led to cytotoxicity or not, the LDH assay was performed on the supernatant from cells exposed to mangostins after 24 h (Figure 1). As expected, the cells dramatically released a pool of LDH when stimulated with LPS and BSO (25.1-fold of the pure UC). It should be noted that cells only pre-incubated with BSO (UC) also abundantly released LDH (29.1-fold). When α- and γ-mangostin were added to the cultures, the release of LDH was significantly decreased (6.6- and 4.8-fold, respectively). It is plausible to assume that the metabolic activation and the decreased cytotoxicity in the presence of mangostins allowed the authors to speculate that the presence of these natural xanthones could even amplify the Nrf2 activation and translocation to counteract the oxidative stress occurrence and, thus, the macrophage activation. In order to demonstrate this speculation, a Western blot analysis was performed (Figure 2). The immunoblotting clearly revealed that Nrf2 translocates into the nuclei in the presence of mangostins under inflammatory conditions, mainly in the presence of the γ derivative. Despite the significant macrophage activation in terms of metabolic activity, it is plausible to assume that mangostins, amplifying the Nrf2 signaling, ameliorate the cell response towards LPS- and BSO-induced oxidative stress and thus counteract cytotoxicity occurrence in these cells. In this light, after having screened all the newly synthesized xanthones derivatives in the same experimental conditions with the cell metabolic activity test (Table 2 and Table 3), the derivatives resembling the cell metabolic activity behavior in the presence of mangostins were chosen for further analyses (namely compounds **5**, **6**, **10**, **16**, **17**, and **27**).

The selected xanthone derivatives underwent LDH release measurements to verify cytotoxicity occurrence. The assay revealed that **17** and **27** were the best compounds capable of decreasing the LDH release from inflamed macrophages (8.6- and 6.8-fold, respectively), and these data are comparable with that obtained in the presence of mangostins (Figure 1). In parallel, Western blot analysis on cell cytosols and nuclei was performed, revealing that **27** was the best compound in activating the Nrf2 translocation, followed by **16** and **10** (Figure 2).

Due to a large number of derivatives and biological data, proper structure–activity relationship (SAR) studies must be extrapolated. Different SAR methods, which relate chemical structure to molecular properties, are frequently used to determine which derivatives should be synthesized based on the information that has been achieved in the optimization procedures. In recent years, matched molecular pairs (MMPs) have become widely used in medicinal chemistry, such as in SAR studies, activity profile analysis, and lead optimization [33]. In fact, a substituent group replacement or a modification in the structure scaffold can cause changes in the physico-chemical properties and activity profile of a molecule. MMPs are defined as pairs of compounds that only differ by a chemical change at a single site. The MMPs transformation can be used to explore changes in a molecule’s dataset activities [34].

When studying SARs, the use of MMPs is useful in identifying structural features that affect activity/potency. The MMP protocol used identified crucial substitutions by identifying all possible MMPs and grouping them by common transformations. The frequency of the transformations indicates the probability that the structural change will give rise to a property variation, and outliers can reveal a means to circumvent the general tendency [35].

**Figure 3:**
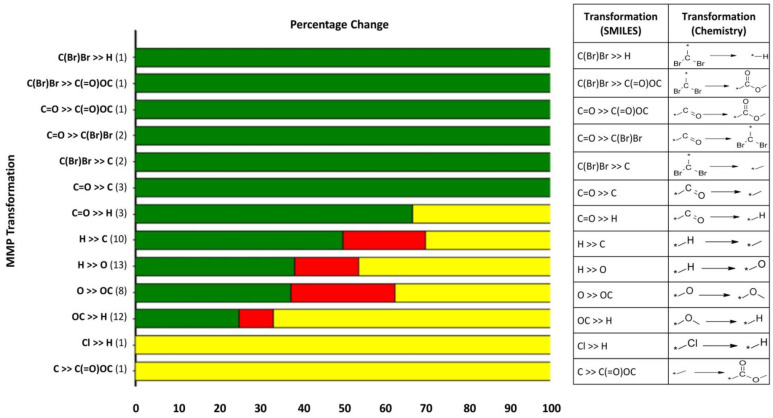
MMPs analysis— bar graph of the MMP transformations versus % that affects activity. Each bar indicates a MMP transformation, and the color coding classifies the activity change: increases activity (green), decreases activity (red), and has neutral effect on activity (yellow). The labels on the left axis indicate the type and number of transformations. Additionally, a table on the right side contains the SMILES transformation and a sketch of the associated reaction. In order to abbreviate the large structures of the tested molecules, * represents the rest of the molecule.

A large library of natural and synthetic xanthone derivatives was studied by MMPs to identify structural trends that could influence activity. Fifty-eight MMPs were found spanning 13 structural substitutions (Figure 3). For each MMP, the activity change was classified as neutral, increased, or decreased. The activity effects are tabulated and sorted to show which MMPs have the largest effects in each category. Six types of substitution were responsible for an increase in activity [C(Br)Br >> H, C(Br)Br >> C(=O)OC, C=O >> C(=O)OC, C=O >> C(Br)Br, C(Br)Br >> C, and C=O > C]; one substitution caused an increase in activity in 66% of the cases, and had no effect in 33% of the cases (C=O >> H); four substitutions caused an increase, a decrease, or had no effect on activity (H >> C, H >> O, O >> OC, OC >> H); and finally, two substitutions did not cause a relevant difference in activity [Cl >> H and C >> C(=O)OC] (Figure 3).

**Figure 4:**
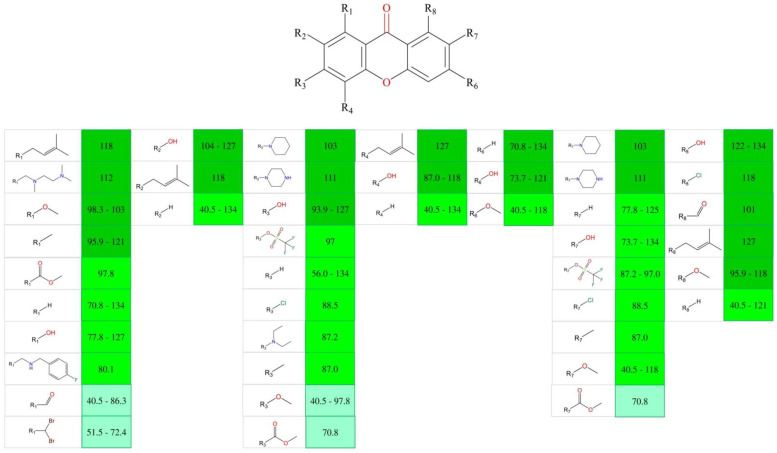
Analysis of activity according to substitutions. Viability at 10 µM after 48 h is represented with a gradient background (darker: higher viability; lighter: lower viability).

Some conclusions could be reached from this MMP analysis, namely the groups that are more favorable for activity. Concerning the molecules studied by MMP analysis, methyl groups always increased activity, and methyl formate groups were typically important for activity (or at least did not affect activity). Conversely, formaldehyde groups were always prejudicial for activity. Other groups had variable behavior according to the group they were substituting. Moreover, -OH groups at positions R2, R3, R6, and/or R8; -OCH_3_ groups at positions R1; prenyl groups at R3, R4, and/or R8; *N*,*N*,*N*′,*N*′-tetramethylpropane-1,3-diamine groups at R1; piperidine and piperazine groups at R3 and/or R7; and -Cl groups at R8 presented the highest activities (Figure 4).

QSAR studies have been used for several years to point out properties of small molecules that are relevant for activity and to forecast the activity of new compounds [36]. Therefore, a QSAR model was built to highlight the descriptors that are relevant to the activity of the tested xanthone derivatives. Furthermore, a QSAR model allows efforts to be directed toward the synthesis of compounds that are more likely to have the desired activity [36]. In this work, a 2D-QSAR model was elaborated using the Comprehensive Descriptors for Structural and Statistical Analysis (CODESSA 2.7.2) software package, which calculates approximately 500 descriptors. The heuristic method performs a pre-selection of descriptors by eliminating descriptors that are not available for each structure, have a small variation in magnitude, are correlated pairwise, and have no statistical significance. The heuristic method is a convenient method for searching for the best set of descriptors, without restrictions on the dataset size [37].

**Figure 5:**
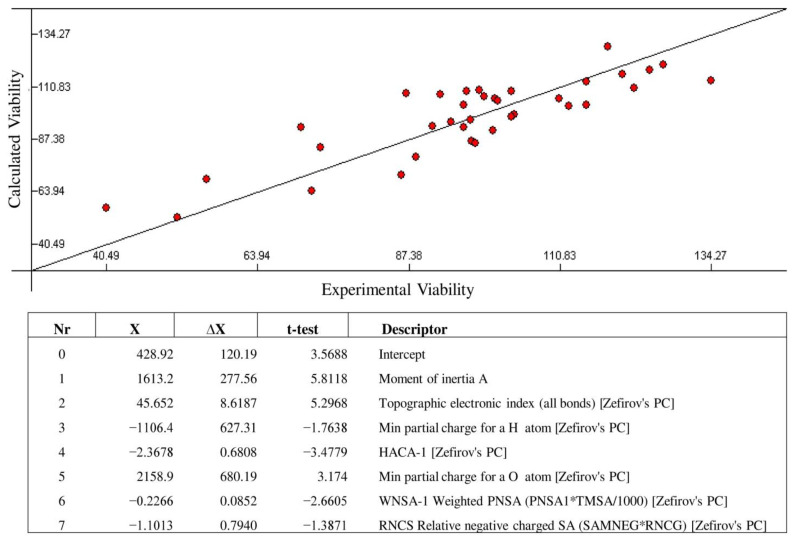
QSAR model obtained with the heuristic method for 36 molecules with the CODESSA software (R^2^ = 0.7346, F = 11.07, S = 11.96). X, ΔX, and *t*-test are the regression coefficients of the linear model, standard errors of the regression coefficient, and the student’s *t*-test significance coefficient of the determination, respectively.

The correlation coefficient (R^2^), standard error (S), and Fisher value (F) measures were used to evaluate the validity of the regression equation [38]. As the rules of QSAR establish that there must be one descriptor for every five molecules used to build the model, seven descriptors were used to build the QSAR equation. The multilinear regression analysis using the heuristic method for 36 compounds in the seven-descriptor model is shown in Figure 5. The compounds are uniformly distributed around the regression line, which indicates that the obtained model has a satisfactory predictive ability.

The best QSAR equation has an R^2^ of 0.7346, a Fisher value of 11.07, and an S of 11.96, which reveals that the proposed model has statistical validity [39]. R^2^ is greater than 0.7, which proves the high strength of the relationship between the QSAR model and the dependent variable [40]; it represents close to 70% of the total variance in the dependent variable (activity) shown by the test compounds. The QSAR model is significant at the 95% level, as shown by the Fisher’s F-test value (F = 11.96), which is larger than the tabulated value (2.36), which is required for a statistically significant model [40]. The standard deviation S displays a small value (s = 11.96), showing that the model is significant and has small variation around the regression line [41]. The reliability of the resulting QSAR model was analyzed using two different types of validation criteria: external validation by using a test set and internal validation by leave-one-out (LOO) cross-validation [42]. The model was able to predict the activity of an external test set with an average difference of 9.00 from the experimental value [43]. Moreover, the cross-validated R^2^ (Q^2^ = 0.5824) from the LOO internal validation process is greater than 0.5 and smaller than the overall R^2^, as anticipated, and the difference between R^2^ and Q^2^ is lower than 0.3, which indicates that the model does not suffer from overfitting [44].

By interpreting the molecular descriptors in the regression model, it is possible to have some insight into structural characteristics that are likely to be responsible for the antioxidant activity of the studied compounds. Seven variables were found to have a significant influence on the potency of the compounds (Figure 5).

Three of the descriptors—moment of inertia A, topographic electronic index, and minimum partial charge for an O atom—have a positive regression coefficient of 1613.2, 45.652, and 2158.9, which means that an increase in these descriptors will lead to an increase in the antioxidant activity of the xanthonic derivatives. On the other hand, minimum partial charge for a H atom, HACA-1, WNSA-1 weighted PNSA, and RNCS relative negative charged SA descriptors have negative regression coefficients of −1106.4, −2.3678, −0.2266, and −1.1013, which reveals that an increase in the values of these descriptors will lead to a decrease in the activity of the molecules.

The moment of Inertia A (*I_A_*) is obtained from the 3D coordinates of the atoms in the given molecule and is defined as the product of the mass times the distance from the axis squared, $I_{A}=\sum_{i}m_{i}r_{ix}^{2}$, where *m_i_* is the atomic mass, and *r_ix_* denotes the distance of the *i*-th atomic nucleus from the main rotational axes, *x*. *I_A_* characterizes the mass distribution in the molecule. The high positive correlation coefficient of the moment of inertia A and the highest *t*-value (*t*-values define the statistical significance of a descriptor) suggest that the orientation behavior in relation to the size of the whole molecule is very important for activity.

The next implicated descriptor is the topographic electronic index, T^E^, which connects submolecular polarity parameters with molecular topography expressed by interatomic distances [45]. This index is calculated as a sum of ratios $\left|q_{i}-q_{j}\right|/r_{i,j}^{2}$ over all the pairs of atoms, both connected and disconnected (*q_i_* and *q_j_* are the corresponding partial charges on atoms *i* and *j* in the pair, whereas *r_i,j_* is the interatomic distance) [46].

Minimal partial charge for a H atom and minimal partial charge for an O atom are electrostatic descriptors related to charge distribution. Electrostatic descriptors consider the electrostatic structure of the molecules characterized by the partial charge distribution or the electronegativity of the atoms. The partial charges in the molecule can be calculated using the method proposed by Zefirov [47], which takes molecular electronegativity as a geometric mean of atomic electronegativity.

HACA1 (hydrogen bonding acceptor ability of the molecule) is a charged partial surface area (CPSA) descriptor that is determined by the equation $\sum_{A}S_{A}\;A\in \;X_{H-acceptor}$ where *S_A_* stands for the solvent-accessible surface area of H-bonding acceptor atoms, selected by threshold charge.

WNSA-1 weighted PNSA (partial negative surface area) is a quantum-chemical descriptor that characterizes molecules by molecular shape and electron distribution and is defined as $\frac{PNSA1\times TMSA}{1000}$, where *PNSA1* is the partial negatively charged molecular surface area, and the *TMSA* is the total molecular surface area. This descriptor is defined based on the total molecular surface area and charge distribution in the molecule, thus indicating the influence of charge distribution on antioxidant activity [48].

RNCS relative negative charged surface area (SAMNEG * RNCG) is an electrostatic descriptor that depends on the distribution of the charges on the molecule. The relative negative charge of the molecule and its surface area can thus influence activity.

The molecular descriptors used in the QSAR model demonstrate that the mechanism underlying the antioxidant activity of xanthones is related to the mass distribution, the polarity of the atoms in relation to their interatomic distances, the electronegativity of hydrogens and oxygens, the hydrogen bonding acceptor ability of the molecule, the molecular shape, and the electron and charge distribution of the molecule. The inspection of the molecular descriptors can result in a better comprehension of the relationship between the structure and activity of xanthones. The QSAR model developed in the present paper may be useful for increasing the awareness of the mechanisms modulating Nrf2 activity.

## 3. Materials and Methods

### 3.1. Synthesis

Xanthones **2**–**11**, **13**–**37**, **39**, and **40** were previously synthesized in our laboratory in the scope of other projects and used without further purification after purity assessment: 1-hydroxy-9*H*-xanthen-9-one (**2**); [15,16,17,19,21], 2-hydroxy-9*H*-xanthen-9-one (**3**); [15,16,17,19,21], 3-hydroxy-9*H*-xanthen-9-one (**4**); [15,16,17,19,21], 4-hydroxy-9*H*-xanthen-9-one (**5**); [15,16,17,19,21], 1-methoxy-9*H*-xanthen-9-one (**6**); [15,19,21,30], 2-methoxy-9*H*-xanthen-9-one (**7**); [15,19,21,30], 4-methoxy-9*H*-xanthen-9-one (**8**); [15,19,21,30], 1,2-dihydroxy-9*H*-xanthen-9-one (**9**); [15,19,25,26,30], 2,3-dihydroxy-9*H*-xanthen-9-one (**10**); [15,19,21,30], 3,4-dihydroxy-9*H*-xanthen-9-one (**11**); [15,19,21,30], 3,6-dihydroxy-9*H*-xanthen-9-one (**13**); [16,21,22,24], 1,2-dimethoxy-9*H*-xanthen-9-one (**14**); [15,19,21,30], 2,3-dimethoxy-9*H*-xanthen-9-one (**15**); [15,19,21,30], 3,4-dimethoxy-9*H*-xanthen-9-one (**16**); [15,19,21,30], 4-hydroxy-3-methoxy-9*H*-xanthen-9-one (**17**); [15,19,21,24,30], 1,3-dihydroxy-2-methoxy-9*H*-xanthen-9-one (**18**); [15,30], 3,4-dihydroxy-9-oxo-9*H*-xanthene-1-carbaldehyde (**19**); [15], 3,4-dimethoxy-9-oxo-9*H*-xanthene-1-carbaldehyde (**20**); [23,27], 3,4,6-trimethoxy-9-oxo-9*H*-xanthene-1-carbaldehyde (**21**); [20,27], 4-hydroxy-3-methoxy-9-oxo-9*H*-xanthene-1-carbaldehyde (**22**); [27,28,29], methyl 3,4,6-trimethoxy-9-oxo-9*H*-xanthene-1-carboxylate (**23**); [27], 3-hydroxy-4-methoxy-9-oxo-9*H*-xanthene-2-carbaldehyde (**24**); [14,15,18], 3,4-dihydroxy-1-methyl-9*H*-xanthen-9-one (**25**); [26,27,30], 3,4-dimethoxy-1-methyl-9*H*-xanthen-9-one (**26**); [20,23,27,28,29,30], 2-chloro-3,4-dimethoxy-1-methyl-9*H*-xanthen-9-one (**27**); [20], 3,4,6-trimethoxy-1-methyl-9*H*-xanthen-9-one (**28**); [20,27,29,30], 1-(dibromomethyl)-3,4-dimethoxy-9*H*-xanthen-9-one (**29**); [23,27,28,29,30], 1-(dibromomethyl)-3,4,6-trimethoxy-9*H*-xanthen-9-one (**30**); [27,29,30], 1,8-dihydroxy-3,6-dimethyl-9*H*-xanthen-9-one (**31**); [26,30], 3,6-dichloro-9*H*-xanthen-9-one (**32**); [30], dimethyl 9-oxo-9*H*-xanthene-3,6-dicarboxylate (**33**); [49], 9-oxo-9*H*-xanthene-3,6-diyl bis(trifluoromethanesulfonate) (**34**); [50], 3,6-di(piperidin-1-yl)-9*H*-xanthen-9-one (**35**); [50], 6-(diethylamino)-9-oxo-9*H*-xanthen-3-yl trifluoromethanesulfonate (**36**); [50], 3,6-di(piperazin-1-yl)-9*H*-xanthen-9-one (**37**); [50], 1-(((3-(dimethylamino)propyl)(methyl)amino)methyl)-3,4-dimethoxy-9*H*-xanthen-9-one HCl (**39**); [27], 1-((5-amino-3,4-dihydroisoquinolin-2(1*H*)-yl)methyl)-3,4-dimethoxy-9*H*-xanthen-9-one (**40**) [23,27]. 1,7-Dihydroxy-9*H*-xanthen-9-one (**12**) was previously isolated from *Cratoxylum maingayi* (Guttiferae) [15]. Xanthone (**1**), α-mangostin, and γ-mangostin (purity ≥98% by HPLC) were purchased from Sigma-Aldrich (Milan, Italy). The purity of compounds **2**–**40** was assessed by HPLC-DAD and was greater than 95%.

### 3.2. Synthesis of 1-(((4-fluorobenzyl)amino)methyl)-3,4-dimethoxy-9H-xanthen-9-one hydrochloride (38)

1-(((4-Fluorobenzyl)amino)methyl)-3,4-dimethoxy-9*H*-xanthen-9-one (**38**) was synthesized, as previously described [27]. An etheric solution was prepared with **38** (purity > 95%) and was cooled at −4 °C. A solution of 2.0 M hydrogen chloride in diethyl ether solution (3 mL) was added to the etheric solution, a white precipitate formed, and the resulting mixture was placed at −4 °C overnight. Dichloromethane (10 mL) was added, the suspension was centrifuged, and the supernatant was placed at −4 °C overnight. The procedure was repeated twice, and the obtained solid was washed with dichloromethane and dried in a desiccator containing phosphorus pentoxide furnishing 1-(((4-fluorobenzyl)amino)methyl)-3,4-dimethoxy-9*H*-xanthen-9-one hydrochloride (**38**).

^1^H NMR (CDCl_3_): *δ* = 8.32 (1H, dd, J = 8.0 and 1.7 Hz, H-8), 7.75 (1H, ddd, J = 8.5, 7.0, and 1.7 Hz, H-6), 7.59 (1H, dd, J = 8.4 and 0.9, H-5), 7.41 (1H, ddd, J = 8.0, 7.1 and 0.9 Hz, H-7), 6.98 (1H, s, H-2), 4.95 (2H, s, H-1′), 4.03 (3H, s, H-4), 4.01 (3H, s, H-3); ^13^C-NMR (CDCl_3_): *δ* = 179.0 (C-6), 156.1 (C-10a), 146.3 (C-12a), 143.2 (C-11a), 134.3 (C-9), 132.4 (C-12), 126.6 (C-7), 123.6 (C-8), 121.5 (C-6a), 118.2 (C-10), 117.9 (C-5), 117.0 (C-4a), 115.4 (C-5a), 76.5 (C-2), 32.6 (C-3), 27.0 (C-4), 21.7 (C-1′).

### 3.3. Cell Culture and Differentiation

Undifferentiated human monocytes (CRL-9855™) were purchased from ATCC^®^ and sub-cultured in RPMI 1640 (Merck, Darmstadt, Germany) supplemented with 10% heat-inactivated fetal bovine serum (FBS), 1% penicillin/streptomycin, and 1% sodium pyruvate (all from Gibco, Invitrogen, Life Technologies, Carlsbad, CA, USA) at 37 °C and 5% CO_2_.

For differentiation into macrophages, the monocytes were seeded in 96 multi-well culture plates (0.5 × 10^4^ cells/well) for the MTT assay and in 150 mm cell culture dishes (0.1 × 10^7^ cells/dish) for the immunoblotting and stimulated with 100 ng/mL of PMA (phorbol-12-myristate-13-acetate, purchased from Merck, Darmstadt, Germany, stock solution 1 mM in DMSO) in complete RPMI for 48 h at 37 °C and 5% CO_2_. After 48 h, a culture of macrophage-like cells was obtained and used for further experimental procedures.

### 3.4. Establishment of the Inflamed Environment and Cell Treatment

After 48 h in RPMI supplemented with 100 ng/mL of PMA, the differentiation medium was discarded and replaced with complete RPMI in the presence of 50 µM BSO (L-buthionine sulfoximine, purchased from Merck, Darmstadt, Germany). The cells were incubated for 18 h at 37 °C and 5% CO_2_. Next, the pre-incubation medium with BSO was discarded and macrophages were exposed to complete RPMI (UC, untreated control), or stimulated with LPS 0.5 µg/mL (lipopolysaccharide from *E. coli*, purchased from Merck, Darmstadt, Germany, stock solution 1 mg/mL in water) and 50 µM BSO and exposed to α- or γ-mangostin (Merck, Darmstadt, Germany) in concentrations of 1–10 µM or xanthone derivatives in concentrations of 1–10 µM for up to 48 h. A pure untreated control (macrophages exposed only to complete RPMI for the entire experimental procedure) was analyzed and used as an internal comparison.

### 3.5. Cell Metabolic Activity (MTT Assay)

At the established time points (24 and 48 h), the exposure media were replaced by 200 µL/well of a solution of 3-(4,5-dimethylthiazol-2-yl)-2,5-diphenyltetrazolium bromide (MTT) 0.5 mg/mL (Merck, Darmstadt, Germany) and processed as elsewhere reported [51]. The optical density in each well was measured using a spectrophotometer (Thermo Fisher Scientific, Waltham, MA, USA) at a wavelength of 540 nm. Each experiment was performed two times in duplicates per experimental condition (n = 4). The optical density readings obtained from treated cell cultures were summarized and normalized to BSO- and LPS-stimulated cell cultures (100%).

### 3.6. Cytotoxicity Assay (LDH Assay)

To quantify cytotoxicity occurrence after 24 h, the CytoTox 96^®^ Non-Radioactive Assay (Promega Corporation, Fitchburg, WI) was performed. The assay quantitatively measures LDH (lactate dehydrogenase), a stable cytosolic enzyme that is released upon cell lysis. Supernatants from 96 well plates used for the MTT assay (50 µL) were analyzed as reported previously [52], and the obtained optical densities were normalized with the ones related to MTT for each sample. The data are the fold increase in the pure UC set as 1. The assay was performed in triplicate for each experimental condition (n = 3).

### 3.7. Protein Extraction

After 24 h, the exposure was stopped by collecting the exposure media and floating cells. Adherent cells were washed with ice-cold PBS, detached with trypsin/5 mM EDTA (EuroClone, Milan, Italy), and collected by centrifugation. The cell pellet was resuspended and washed twice in ice-cold PBS. Next, nuclear and cytosolic extracts were separated using different lysis buffers as described [53]. The protein concentration was determined using a bicinchoninic acid assay (QuantiPro^TM^ BCA Assay kit for 0.5–30 mg/mL protein, Merck, Darmstadt, Germany) following the manufacturer’s instructions. The assay was performed in triplicate for each experimental condition (n = 3).

### 3.8. Immunoblotting (Western Blot)

Proteins (20 µg per lane) were first separated on a 10% sodium dodecyl sulfate-polyacrylamide gel by electrophoresis (SDS-PAGE) and transferred to a nitrocellulose membrane in SDS buffer (25 mM Tris HCl, 192 mM glycine, 20% methanol, pH 8.3) at 350 mA for 120 min. Nitrocellulose membranes were then blocked in 5% of non-fat milk or 5% of BSA, 10 mmol/L Tris pH 7.5, 100 mM NaCl, 0.1% Tween 20 and probed overnight at 4 °C under gentle shaking with rabbit polyclonal anti-Nrf2 (primary antibody dilution 1:750) purchased from Santa Cruz Biotechnology (Santa Cruz, CA, USA), mouse monoclonal anti-β-actin (primary antibody dilution 1:5000), and mouse monoclonal anti-laminin A/C (primary antibody dilution 1:1000) all purchased from Merck, Darmstadt, Germany. Afterward, membranes were incubated in the presence of specific IgG horseradish peroxidase (HRP)-conjugated secondary antibodies. Immunoreactive bands were identified using the ECL detection system (LiteAblot Extend Chemiluminescent Substrate, EuroClone S.p.A., Milan, Italy) and analyzed by densitometry. Densitometric values, expressed as integrated optical intensity (IOI), were estimated in the CHEMIDOC XRS system using the QuantiOne 1-D analysis software (BIORAD, Richmond, CA, USA). The values obtained were normalized based on densitometric values of internal cytosolic actin or nuclear laminin and were expressed as relative expressions of Nrf2 (n = 3).

### 3.9. Structure–Activity Analysis Using Matched Molecular Pairs (MMPs)

The MMPs were calculated using the 42 molecules (test molecules and controls) and the MMP analysis of activity effects protocol in Discovery Studio Biovia 2016. The activity was set as the viability at 10 µM after 48 h. The minimum frequency was set to 1, and the activity change threshold was set to 10. A bar graph of the transformations that most frequently changed the activity was obtained.

### 3.10. Quantitative Structure–Activity Relationship (QSAR)

The forty test xanthone derivatives and the two xanthone controls (α- and γ-mangostin) were used to build the QSAR model using the experimental data obtained from the in vitro studies (viability at 10 µM after 48 h). Viability was selected as a dependent variable in the QSAR analysis. The 42 molecules were randomly distributed into a training set (36 molecules) and a test set (6 molecules). CODESSA software (version 2.7.10, University of Florida, Gainesville, FL, USA) was used to calculate hundreds of constitutional, topological, geometrical, electrostatic, quantum-chemical, and thermodynamical molecular descriptors [54]. The heuristic multilinear regression methodology was chosen to perform a complete search for the best multilinear correlations with a multitude of descriptors of the training set [37]. The 2D-QSAR model with the best square of the correlation coefficient (R^2^), F-test (F), and squared standard error (S^2^) was selected. The final model was further validated using the test set and leave-one-out (LOO) internal validation.

## 4. Conclusions

A library of forty newly synthesized and natural xanthone derivatives were evaluated in vitro on human macrophages under pro-inflammatory conditions to select the greatest-performing derivative for oxidative stress counteraction in terms of cytotoxicity and Nrf2 nuclear translocation. With this aim, compounds **5**, **6**, **10**, **16**, **17**, and **27** were selected, disclosing that **27** was the best compound for decreasing cytotoxicity and enhancing Nrf2 translocation and, thus, activation. Moreover, a QSAR study revealed that topological, geometrical, electrostatic, charged partial surface area, and quantum-chemical descriptors were implicated in the antioxidant activity of the tested xanthones.

## Figures and Tables

**Table 1:**
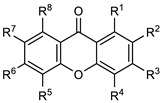
Structure of xanthones **1**–**40**.

Compound	R^1^	R^2^	R^3^	R^4^	R^5^	R^6^	R^7^	R^8^
**1**	H	H	H	H	H	H	H	H
**2**	OH	H	H	H	H	H	H	H
**3**	H	OH	H	H	H	H	H	H
**4**	H	H	OH	H	H	H	H	H
**5**	H	H	H	OH	H	H	H	H
**6**	OCH_3_	H	H	H	H	H	H	H
**7**	H	OCH_3_	H	H	H	H	H	H
**8**	H	H	H	OCH_3_	H	H	H	H
**9**	OH	OH	H	H	H	H	H	H
**10**	H	OH	OH	H	H	H	H	H
**11**	H	H	OH	OH	H	H	H	H
**12**	OH	H	H	H	H	H	OH	H
**13**	H	H	OH	H	H	OH	H	H
**14**	OCH_3_	OCH_3_	H	H	H	H	H	H
**15**	H	OCH_3_	OCH_3_	H	H	H	H	H
**16**	H	H	OCH_3_	OCH_3_	H	H	H	H
**17**	H	H	OCH_3_	OH	H	H	H	H
**18**	OH	OCH_3_	OH	H	H	H	H	H
**19**	CHO	H	OH	OH	H	H	H	H
**20**	CHO	H	OCH_3_	OCH_3_	H	H	H	H
**21**	CHO	H	OCH_3_	OCH_3_	H	OCH_3_	H	H
**22**	CHO	H	OCH_3_	OH	H	H	H	H
**23**	COOCH_3_	H	OCH_3_	OCH_3_	H	OCH_3_	H	H
**24**	H	CHO	OH	OCH_3_	H	H	H	H
**25**	CH_3_	H	OH	OH	H	H	H	H
**26**	CH_3_	H	OCH_3_	OCH_3_	H	H	H	H
**27**	CH_3_	Cl	OCH_3_	OCH_3_	H	H	H	H
**28**	CH_3_	H	OCH_3_	OCH_3_	H	OCH_3_	H	H
**29**	CHBr_2_	H	OCH_3_	OCH_3_	H	H	H	H
**30**	CHBr_2_	H	OCH_3_	OCH_3_	H	OCH_3_	H	H
**31**	OH	H	CH_3_	H	H	CH_3_	H	OH
**32**	H	H	Cl	H	H	Cl	H	H
**33**	H	H	CO_2_CH_3_	H	H	CO_2_CH_3_	H	H
**34**	H	H	OTf	H	H	OTf	H	H
**35**	H	H	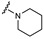	H	H	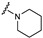	H	H
**36**	H	H	N(CH_3_)_2_	H	H	N(CH_3_)_2_	H	H
**37**	H	H	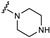	H	H	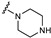	H	H
**38**	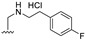	H	OCH_3_	OCH_3_	H	H	H	H
**49**	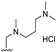	H	OCH_3_	OCH_3_	H	H	H	H
**40**	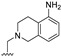	H	OCH_3_	OCH_3_	H	H	H	H
**α-mangostin**	OH	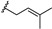	OH	H	H	OH	OCH_3_	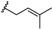
**γ-mangostin**	OH	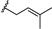	OH	H	H	OH	OH	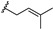

**Table 2:** Cell metabolic activity percentages in the presence of compounds **1**–**40** at 1 µM obtained from the MTT assay.

Compound (1 µM)	Viability 24 h (% LPS)	Viability 48 h (% LPS)
**1**	81.3 ± 5.1 ^d,g^	118.7 ± 23.1
**2**	77.9 ± 2.6 ^a,e,h^	98.6 ± 25.5
**3**	116.5 ± 28.9	137.1 ± 16.5
**4**	123.4 ± 4.1	130.8 ± 6.7 ^a^
**5**	151.7 ± 5.2 ^a^	144.6 ± 7.0 ^b^
**6**	168.7 ± 5.2 ^c^	147.4 ± 3.1 ^b^
**7**	110.8 ± 0.7	105.9 ± 15.0
**8**	97.6 ± 17.0	100.6 ± 18.8
**9**	135.2 ± 39.6 ^b^	147.2 ± 6.4
**10**	131.8 ± 3.3 ^a^	127.1 ± 5.3 ^a^
**11**	119.3 ± 7.6	104.7 ± 17.8 ^g^
**12**	92.6 ± 5.0 ^f,i^	107.3 ± 15.4
**13**	114.7 ± 0.6	101.4 ± 12.8 ^d,g^
**14**	122.3 ± 1.8	102.0 ± 11.6 ^g^
**15**	109.5 ± 1.9 ^h^	108.9 ± 3.0
**16**	111.3 ± 10.0 ^g^	130.9 ± 1.9
**17**	143.4 ± 14.5 ^a^	123.5 ± 5.3
**18**	104.8 ± 12.0 ^d,h^	109.7 ± 5.9
**19**	105.4 ± 4.9 ^d,h^	111.8 ± 2.5
**20**	134.5 ± 2.8 ^a^	101.3 ± 2.4 ^d,g^
**21**	90.8 ± 0.5 ^f,i^	93.3 ± 6.6 ^e,i^
**22**	93.4 ± 0.4 ^f,i^	107.6 ± 11.1
**23**	112.7 ± 3.6 ^g^	91.4 ± 6.0 ^e,i^
**24**	102.0 ± 11.4 ^e,i^	83.7 ± 17.1 ^f,i^
**25**	110.7 ± 8.4 ^h^	99.9 ± 15.0 ^d,h^
**26**	100.8 ± 7.0 ^f,h^	99.9 ± 7.2
**27**	101.6 ± 16.9 ^e,h^	124.8 ± 8.4 ^a^
**28**	112.5 ± 2.0 ^g^	108.8 ± 8.1
**29**	89.4 ± 1.5 ^f,i^	98.4 ± 2.2 ^d,h^
**30**	104.7 ± 1.5 ^e,i^	108.4 ± 7.3
**31**	104.0 ± 2.7 ^e,i^	105.5 ± 10.6 ^g^
**32**	96.6 ± 8.8 ^f,i^	94.8 ± 3.4 ^e,i^
**33**	98.0 ± 2.9 ^f,i^	82.8 ± 9.0 ^f,i^
**34**	87.0 ± 5.6 ^f,i^	85.5 ± 3.6 ^f,i^
**35**	111.8 ± 3.5 ^f,i^	115.3 ± 2.2 ^a,h^
**36**	117.1 ± 3.8 ^a,f,i^	100.9 ± 2.7 ^e,h^
**37**	112.9 ± 0.3 ^f,i^	103.3 ± 0.1
**38**	90.0 ± 4.2 ^b,f,i^	84.5 ± 11.5 ^f,i^
**39**	96.7 ± 0.5 ^f,i^	83.5 ± 7.0 ^f,i^
**40**	82.9 ± 1.4 ^f,i^	85.3 ± 2.0 ^a,f,i^
**α-mangostin**	135.4 ± 0.2 ^b^	120.3 ± 1.4 ^a^
**γ-mangostin**	140.2 ± 1.4 ^c^	123.2 ± 3.5 ^a^

% LPS = LPS set as 100%; LPS = 18 h of pre-incubation with BSO 50 µM then 24 or 48 h with BSO 50 µM + LPS 0.5 µg/mL; compounds 1 µM = 18 h of pre-incubation with BSO 50 µM then 24 or 48 h with BSO 50 µM + LPS 0.5 µg/mL + compounds 1 µM. a = *p* < 0.05, b = *p* < 0.005, and c = *p* < 0.0005 between compounds and LPS; d = *p* < 0.05, e = *p* < 0.005, and f = *p* < 0.0005 between compounds and α-mangostin at the same concentration; g = *p* < 0.05, h = *p* < 0.005, and i = *p* < 0.0005 between compounds and γ-mangostin at the same concentration.

**Table 3:** Cell metabolic activity percentages in the presence of compounds **1**–**40** at 10 µM obtained from the MTT assay.

Compound (10 µM)	Viability 24 h (% LPS)	Viability 48 h (% LPS)
**1**	108.0 ± 0.3 ^m^	100.7 ± 7.5
**2**	81.2 ± 0.4	77.7 ± 15.8 ^g^
**3**	112.4 ± 19.8	122.3 ± 1.3
**4**	117.5 ± 6.8	99.0 ± 3.5 ^m^
**5**	144.1 ± 19.3 ^a^	96.3 ± 2.7 ^n^
**6**	123.8 ± 5.5 ^n^	102.9 ± 5.8 ^n^
**7**	133.2 ± 11.3 ^a^	95.8 ± 1.7
**8**	92.8 ± 10.5	97.0 ± 1.3
**9**	127.2 ± 5.7	124.7 ± 1.8
**10**	134.3 ± 9.3 ^a^	134.2 ± 4.6 ^a^
**11**	115.7 ± 2.9	91.9 ± 8.0
**12**	100.1 ± 3.9 ^i^	103.7 ± 16.1
**13**	111.9 ± 1.1	93.8 ± 3.9
**14**	111.2 ± 2.4	98.3 ± 0.1
**15**	98.3 ± 10.1	101.1 ± 0.2
**16**	116.6 ± 10.6	103.3 ± 3.7
**17**	111.2 ± 5.9	92.3 ± 15.0
**18**	106.1 ± 14.2	115.0 ± 5.3
**19**	116.4 ± 1.9	73.7 ± 4.2 ^m,e,h^
**20**	86.0 ± 2.3 ^n,d,h^	56.0 ± 6.4 ^a,m,f,i^
**21**	51.8 ± 9.9 ^a,m,f,i^	40.4 ± 11.3 ^b,m,f,i^
**22**	100.1 ± 0.1	86.2 ± 3.7 ^g^
**23**	111.8 ± 6.1	97.7 ± 16.1
**24**	101.9 ± 0.9	100.4 ± 1.3
**25**	101.6 ± 2.0	120.5 ± 7.1
**26**	114.0 ± 7.9	115.0 ± 8.2
**27**	102.0 ± 1.3	118.0 ± 3.0 ^a^
**28**	100.8 ± 2.7	95.9 ± 11.3
**29**	77.5 ± 3.3 ^a,f,i^	72.3 ± 1.3 ^c,r,e,i^
**30**	53.0 ± 6.6 ^b,r,f,i^	51.5 ± 1.3 ^c,r,f,i^
**31**	91.1 ± 1.1 ^h^	87.0 ± 8.2 ^g^
**32**	92.4 ± 3.7 ^g^	88.5 ± 2.5
**33**	88.3 ± 10.7 ^g^	70.7 ± 7.1 ^a,e,i^
**34**	94.6 ± 7.1 ^g^	96.9 ± 8.6
**35**	109.0 ± 0.8 ^h^	103.3 ± 0.7 ^h^
**36**	91.7 ± 2.1 ^n,f,i^	87.1 ± 7.6 ^f,i^
**37**	92.8 ± 0.1 ^m,f,i^	110.6 ± 3.4 ^h^
**38**	82.2 ± 0.7 ^a,f,i^	80.0 ± 7.1 ^f,i^
**39**	91.8 ± 2.2 ^a,f,i^	112.2 ± 1.2 ^a,h^
**40**	76.86 ± 1.7 ^m,f,i^	91.0 ± 1.4 ^n,f,i^
**α-mangostin**	115.1 ± 0.1	118.2 ± 1.3 ^a^
**γ-mangostin**	120.1 ± 0.6 ^a,m^	126.9 ± 0.7 ^b^

% LPS = LPS set as 100%; LPS = 18 h of pre-incubation with BSO 50 µM then 24 or 48 h with BSO 50 µM + LPS 0.5 µg/mL; compounds 10 µM = 18 h of pre-incubation with BSO 50 µM then 24 or 48 h with BSO 50 µM + LPS 0.5 µg/mL + compounds 10 µM. a = *p* < 0.05, b = *p* < 0.005, and c = *p* < 0.0005 between compounds and LPS; d = *p* < 0.05, e = *p* < 0.005, and f = *p* < 0.0005 between compounds and α-mangostin at the same concentration; g = *p* < 0.05, h = *p* < 0.005, and i = *p* < 0.0005 between compounds and γ-mangostin at the same concentration; m = *p* < 0.05, n = *p* < 0.005, and r = *p* < 0.0005 between the same compound at 10 µM and 1 µM.

## Data Availability

Data are contained within the article.

## References

[1] Huang Q., Wang Y., Wu H., Yuan M., Zheng C., Xu H. (2021). Xanthone Glucosides: Isolation, Bioactivity and Synthesis. Molecules.

[2] Pinto M.M.M., Palmeira A., Fernandes C., Resende D.I.S.P., Sousa E., Cidade H., Tiritan M.E., Correia-da-Silva M., Cravo S. (2021). From Natural Products to New Synthetic Small Molecules: A Journey through the World of Xanthones. Molecules.

[3] Gunter N.V., Teh S.S., Lim Y.M., Mah S.H. (2020). Natural Xanthones and Skin Inflammatory Diseases: Multitargeting Mechanisms of Action and Potential Application. Front. Pharmacol..

[4] Yadav V.P., Shukla A., Choudhury S., Singh R., Anand M., Prabhu S.N. (2022). IL1β/ TNFα/COX-2/VEGF axis responsible for effective healing potential of C-glucoside xanthone (mangiferin) based ointment in immunocompromised rats. Cytokine.

[5] Wang M.-H., Zhang K.-J., Gu Q.-L., Bi X.-L., Wang J.-X. (2017). Pharmacology of mangostins and their derivatives: A comprehensive review. Chin. J. Nat. Med..

[6] Ibrahim M.Y., Hashim N.M., Mariod A.A., Mohan S., Abdulla M.A., Abdelwahab S.I., Arbab I.A. (2016). α-Mangostin from *Garcinia mangostana* Linn: An updated review of its pharmacological properties. Arab. J. Chem..

[7] Chen G., Li Y., Wang W., Deng L. (2018). Bioactivity and pharmacological properties of α-mangostin from the mangosteen fruit: A review. Expert. Opin. Ther. Pat..

[8] Meylina L., Muchtaridi M., Joni I.M., Mohammed A.F.A., Wathoni N. (2021). Nanoformulations of α-Mangostin for Cancer Drug Delivery System. Pharmaceutics.

[9] Bumrungpert A., Kalpravidh R.W., Chuang C.-C., Overman A., Martinez K., Kennedy A., McIntosh M. (2010). Xanthones from mangosteen inhibit inflammation in human macrophages and in human adipocytes exposed to macrophage-conditioned media. J. Nutr..

[10] Kobayashi E.H., Suzuki T., Funayama R., Nagashima T., Hayashi M., Sekine H., Tanaka N., Moriguchi T., Motohashi H., Nakayama K. (2016). Nrf2 suppresses macrophage inflammatory response by blocking proinflammatory cytokine transcription. Nat. Commun..

[11] Schweikl H., Gallorini M., Pöschl G., Urmann V., Petzel C., Bolay C., Hiller K.-A., Cataldi A., Buchalla W. (2018). Functions of transcription factors NF-κB and Nrf2 in the inhibition of LPS-stimulated cytokine release by the resin monomer HEMA. Dent. Mater..

[12] Fang Y., Su T., Qiu X., Mao P., Xu Y., Hu Z., Zhang Y., Zheng X., Xie P., Liu Q. (2016). Protective effect of alpha-mangostin against oxidative stress induced-retinal cell death. Sci. Rep..

[13] Fu T., Li H., Zhao Y., Cai E., Zhu H., Li P., Liu J. (2018). Hepatoprotective effect of α-mangostin against lipopolysaccharide/d-galactosamine-induced acute liver failure in mice. Biomed. Pharmacother..

[14] Fernandes E.G.R., Silva A.M.S., Cavaleiro J.A.S., Silva F.M., Borges M.F.M., Pinto M.M.M. (1998). 1H and 13C NMR spectroscopy of mono-, di-, tri- and tetrasubstituted xanthones. Magn. Reson. Chem..

[15] Pedro M., Cerqueira F., Sousa M.E., Nascimento M.S.J., Pinto M. (2002). Xanthones as inhibitors of growth of human cancer cell lines and Their effects on the proliferation of human lymphocytes In Vitro. Bioorg. Med. Chem..

[16] Pinto E., Afonso C., Duarte S., Vale-Silva L., Costa E., Sousa M.E., Pinto M. (2011). Antifungal Activity of Xanthones: Evaluation of their Effect on Ergosterol Biosynthesis by High-performance Liquid Chromatography. Chem. Biol. Drug Des..

[17] Leão M., Pereira C., Bisio A., Ciribilli Y., Paiva A.M., Machado N., Palmeira A., Fernandes M.X., Sousa E., Pinto M. (2013). Discovery of a new small-molecule inhibitor of p53–MDM2 interaction using a yeast-based approach. Biochem. Pharmacol..

[18] Bessa L.J., Palmeira A., Gomes A.S., Vasconcelos V., Sousa E., Pinto M., da Costa P.M. (2015). Synergistic Effects Between Thioxanthones and Oxacillin Against Methicillin-Resistant *Staphylococcus aureus*. Microb. Drug Resist..

[19] Cidade H., Rocha V., Palmeira A., Marques C., Tiritan M.E., Ferreira H., Lobo J.S., Almeida I.F., Sousa M.E., Pinto M. (2020). In silico and in vitro antioxidant and cytotoxicity evaluation of oxygenated xanthone derivatives. Arab. J. Chem..

[20] Resende D.I.S.P., Pereira-Terra P., Inácio Â.S., da Costa P.M., Pinto E., Sousa E., Pinto M.M.M. (2018). Lichen Xanthones as Models for New Antifungal Agents. Molecules.

[21] Urbatzka R., Freitas S., Palmeira A., Almeida T., Moreira J., Azevedo C., Afonso C., Correia-Da-Silva M., Sousa M.E., Pinto M. (2018). Lipid reducing activity and toxicity profiles of a library of polyphenol derivatives. Eur. J. Med. Chem..

[22] Alves A., Correia-Da-Silva M., Nunes C., Campos J., Sousa E., Silva P.M., Bousbaa H., Rodrigues F., Ferreira D., Costa P.C. (2019). Discovery of a New Xanthone against Glioma: Synthesis and Development of (Pro)liposome Formulations. Molecules.

[23] Lemos A., Gomes A.S., Loureiro J.B., Brandão P., Palmeira A., Pinto M.M.M., Saraiva L., Sousa M.E. (2019). Synthesis, Biological Evaluation, and In Silico Studies of Novel Aminated Xanthones as Potential p53-Activating Agents. Molecules.

[24] Martins E., Silva V., Lemos A., Palmeira A., Puthongking P., de Sousa M.E., Rocha-Pereira C., Ghanem C.I., Carmo H., Remião F. (2019). Newly Synthesized Oxygenated Xanthones as Potential P-Glycoprotein Activators: In Vitro, Ex Vivo, and In Silico Studies. Molecules.

[25] Silva V., Cerqueira F., Nazareth N., Medeiros R., Sarmento A., Sousa E., Pinto M. (2019). 1,2-Dihydroxyxanthone: Effect on A375-C5 Melanoma Cell Growth Associated with Interference with THP-1 Human Macrophage Activity. Pharmaceuticals.

[26] Resende D.I.S.P., Almeida M.C., Maciel B., Carmo H., Lobo J.S., Pozzo C.D., Cravo S.M., Rosa G.P., Kane-Pagès A., Barreto M.D.C. (2020). Efficacy, Stability, and Safety Evaluation of New Polyphenolic Xanthones Towards Identification of Bioactive Compounds to Fight Skin Photoaging. Molecules.

[27] Resende D.I.S.P., Pereira-Terra P., Moreira J., Freitas-Silva J., Lemos A., Gales L., Pinto E., de Sousa M.E., Da Costa P.M., Pinto M.M.M. (2020). Synthesis of a Small Library of Nature-Inspired Xanthones and Study of Their Antimicrobial Activity. Molecules.

[28] Silva V., Gil Martins E., Rocha-Pereira C., Lemos A., Palmeira A., Puthongking P., Sousa E., Bastos M.D.L., Remião F., Silva R. (2020). Oxygenated xanthones as P-glycoprotein modulators at the intestinal barrier: In vitro and docking studies. Med. Chem. Res..

[29] Resende D.I.S.P., Almeida J.R., Pereira S., Campos A., Lemos A., Plowman J.E., Thomas A., Clerens S., Vasconcelos V., Pinto M. (2021). From Natural Xanthones to Synthetic C-1 Aminated 3,4-Dioxygenated Xanthones as Optimized Antifouling Agents. Mar. Drugs.

[30] Rosa G., Palmeira A., Resende D., Almeida I., Kane-Pagès A., Barreto M., Sousa E., Pinto M. (2020). Xanthones for melanogenesis inhibition: Molecular docking and QSAR studies to understand their anti-tyrosinase activity. Bioorg. Med. Chem..

[31] Soidinsalo O., Wähälä K. (2007). Aromatic Chlorination with Thionyl Chloride. Applications in the Synthesis of Chlorinated Isoflavones. Phosphorus Sulfur Silicon Relat. Elem..

[32] Schweikl H., Birke M., Gallorini M., Petzel C., Bolay C., Waha C., Hiller K.-A., Buchalla W. (2021). HEMA-induced oxidative stress inhibits NF-κB nuclear translocation and TNF release from LTA- and LPS-stimulated immunocompetent cells. Dent. Mater..

[33] Tyrchan C., Evertsson E. (2016). Matched Molecular Pair Analysis in Short: Algorithms, Applications and Limitations. Comput. Struct. Biotechnol. J..

[34] Griffen E., Leach A.G., Robb G.R., Warner D.J. (2011). Matched Molecular Pairs as a Medicinal Chemistry Tool. J. Med. Chem..

[35] Ding X., Cui C., Wang D., Zhao J., Zheng M., Luo X., Jiang H., Chen K. (2020). Bioactivity Prediction Based on Matched Molecular Pair and Matched Molecular Series Methods. Curr. Pharm. Des..

[36] Arodz T., Galvez J. (2006). Computational Methods in Developing Quantitative Structure-Activity Relationships (QSAR): A Review. Comb. Chem. High Throughput Screen..

[37] Ćwik J., Koronacki J., Polkowski L., Skowron A. A Heuristic Method of Model Choice for Nonlinear Regression. Rough Sets and Current Trends in Computing, Proceedings of the First International Conference, RSCTC’98, Warsaw, Poland, 22–26 June 1998.

[38] Dunn W.J., Hopfinger A.J. (2002). 3D QSAR of Flexible Molecules using Tensor Representation.

[39] Kubinyi H. (1993). QSAR: Hansch Analysis and Related Approaches.

[40] Veerasamy R., Rajak H., Jain A., Sivadasan S., Varghese C.P., Agrawal R.K. (2011). Validation of QSAR Models—Strategies and Importance. Int. J. Drug Discov..

[41] Alexander D.L.J., Tropsha A., Winkler D.A. (2015). Beware of R2: Simple, Unambiguous Assessment of the Prediction Accuracy of QSAR and QSPR Models. J. Chem. Inform. Mod..

[42] Liu P., Long W. (2009). Current Mathematical Methods Used in QSAR/QSPR Studies. Int. J. Mol. Sci..

[43] Gramatica P., Reisfeld B., Mayeno A.N. (2013). On the Development and Validation of QSAR Models. Computational Toxicology: Volume II.

[44] Golbraikh A., Shen M., Xiao Z., Xiao Y.-D., Lee K.-H., Tropsha A. (2003). Rational selection of training and test sets for the development of validated QSAR models. J. Comput. Aided Mol. Des..

[45] Devillers J., Balaban A.T. (2018). Topological Indices and Related Descriptors in QSAR and QSPR.

[46] Katritzky A.R., Gordeeva E.V. (1993). Traditional topological indices vs electronic, geometrical, and combined molecular descriptors in QSAR/QSPR research. J. Chem. Inf. Comput. Sci..

[47] Katritzky A.R., Tamm T., Wang Y., Sild S., Karelson M. (1999). QSPR Treatment of Solvent Scales. J. Chem. Inf. Comput. Sci..

[48] Todeschini R., Consonni V. (2009). Methods and Principles in Medicinal Chemistry—Molecular Descriptors for Chemoinformatics.

[49] Carson J.R., Codd E., Razler C.M., Works A., McDonnell M., McNally J.J. (2009). Tricyclic-Bridged Piperidinylidene Derivatives as 8-Opioid Modulators. U.S. Patent.

[50] Wu L., Burgess K. (2008). Synthesis and Spectroscopic Properties of Rosamines with Cyclic Amine Substituents. J. Org. Chem..

[51] Berrino E., Carradori S., Angeli A., Carta F., Supuran C., Guglielmi P., Coletti C., Paciotti R., Schweikl H., Maestrelli F. (2021). Dual Carbonic Anhydrase IX/XII Inhibitors and Carbon Monoxide Releasing Molecules Modulate LPS-Mediated Inflammation in Mouse Macrophages. Antioxidants.

[52] Gallorini M., Zara S., Ricci A., Mangano F.G., Cataldi A., Mangano C. (2021). The Open Cell Form of 3D-Printed Titanium Improves Osteconductive Properties and Adhesion Behavior of Dental Pulp Stem Cells. Materials.

[53] Gallorini M., Petzel C., Bolay C., Hiller K.-A., Cataldi A., Buchalla W., Krifka S., Schweikl H. (2015). Activation of the Nrf2-regulated antioxidant cell response inhibits HEMA-induced oxidative stress and supports cell viability. Biomaterials.

[54] Katritsky A., Karelson M., Lobanov V., Dennington R., Keith T. (2004). CODESSA 2.7. 10.

